# BMI Levels Among Adolescents Aged 10-19 Years Attending Dubai Health Facilities in 2023

**DOI:** 10.7759/cureus.115855

**Published:** 2026-09-06

**Authors:** Marwah Al Shmanee, Fatimah Resen, Ayesha Almarzooqi, Zekeria Kerem, Maha Abdo, Ayesha Almheiri

**Affiliations:** 1 Obstetrics and Gynaecology, Dubai Health, Dubai, ARE; 2 Graduate Medical Education, Mohammed Bin Rashid University of Medicine and Health Sciences, Dubai, ARE; 3 Family Medicine, SEHA Sheikh Khalifa Medical City, Abu Dhabi, ARE; 4 Family Medicine, Dubai Health, Dubai, ARE

**Keywords:** adolescents, bmi measurement, body mass index, malnutrition, nutritional status, obesity, overweight, public health, retrospective study, underweight

## Abstract

Introduction

Adolescence is the phase of life between childhood and adulthood, from ages 10 to 19 years. It is a critical developmental period characterized by rapid physical, psychological, and behavioral changes. Body mass index (BMI) is an important indicator of nutritional status and overall health, with both underweight and abnormal BMI levels associated with short- and long-term health consequences. This study aimed to assess BMI screening rate and BMI levels among adolescents aged 10-19 years attending Dubai Health facilities in 2023.

Methods

A retrospective descriptive study was conducted among adolescents aged 10-19 years who attended Dubai Health facilities between January 1 and December 31, 2023. Data were extracted from the EPIC electronic medical record system (Epic Systems Corporation, Verona, Wisconsin, United States) using the Slicer Dicer tool. BMI status was categorized according to World Health Organization (WHO) age- and sex-specific percentile criteria. BMI-for-age categories were extracted directly from the EPIC Slicer Dicer system without manual recalculation. Data analysis was performed using Microsoft Excel (Microsoft Corporation, Redmond, Washington, United States), and results were presented using descriptive statistics.

Results

A total of 496,498 adolescents were included in the study. Of these, 40,726 (8.2%) had documented BMI measurements, while 455,772 (91.8%) did not. Among adolescents with recorded BMI, 2,974 (7.3%) were underweight, 20,442 (50.2%) had normal BMI, 7,576 (18.6%) were overweight, and 9,734 (23.9%) were obese.

Conclusion

A significant proportion of adolescents did not undergo BMI screening in healthcare facilities, highlighting a major gap in routine health assessment. Among those assessed, a considerable proportion had abnormal BMI, including both underweight and overweight/obesity. These findings emphasize the need to strengthen BMI screening programs, promote early identification of nutritional abnormalities, and implement targeted public health interventions to improve adolescent health outcomes in Dubai.

## Introduction

Adolescence is the phase of life between childhood and adulthood, from ages 10 to 19 years [1]. It is a critical developmental period characterized by rapid physical growth, hormonal changes, and psychological maturation. During this stage, individuals develop behavioral patterns related to diet and physical activity, many of which may persist into adulthood and influence long-term health outcomes.

Body mass index (BMI) is a widely used indicator for assessing nutritional status and body fatness, calculated as weight in kilograms divided by height in meters squared. According to the World Health Organization (WHO) growth reference for children and adolescents, BMI is classified using age- and sex-specific percentile cut-offs as underweight (<3rd percentile), normal weight (≥3rd to ≤85th percentile), overweight (>85th to ≤97th percentile), and obesity (>97th percentile) [2]. Despite known limitations, BMI remains a practical and widely accepted tool for population-level screening in both clinical and public health settings.

Abnormal BMI during adolescence is associated with significant health consequences. Underweight status may reflect malnutrition and is associated with impaired growth, delayed development, and increased risk of anemia and menstrual disorders in adolescent girls [3]. On the other hand, overweight and obesity among adolescents are increasingly prevalent worldwide [4] and are strongly associated with metabolic complications, including type 2 diabetes mellitus, hypertension, dyslipidemia, and polycystic ovary syndrome [5,6]. Importantly, adolescent obesity is a strong predictor of obesity in adulthood, leading to increased long-term morbidity and mortality [5].

BMI is influenced by multiple factors, including dietary habits, physical activity, socioeconomic status, cultural practices, and parental behaviors. Environmental and lifestyle factors also play an important role in determining weight status during adolescence. Early identification of abnormal BMI is therefore essential to allow timely intervention and prevention of long-term health consequences.

In the United Arab Emirates, adolescents represent a diverse population with varying cultural and lifestyle backgrounds. Understanding BMI patterns in this group is essential for guiding public health strategies aimed at promoting healthy behaviors and reducing the burden of both undernutrition and obesity. This study aimed to identify the distribution of BMI levels and the rate of BMI screening among adolescents aged 10-19 years attending Dubai Health facilities in 2023.

This work was previously presented as an e-poster at The Royal College of Obstetricians and Gynecologists (RCOG) World Congress, London, United Kingdom, held on June 23-25, 2025.

## Materials and methods

Study design, setting, and population

This was a retrospective descriptive study conducted across the Dubai Health network, including six hospitals, 26 health centers and ambulatory care units, and 21 medical fitness centers, with the analysis encompassing all accessible electronic medical record data relevant to the adolescent population. The study population included all adolescents aged 10-19 years who attended these facilities between January 1, 2023, and December 31, 2023. No exclusion criteria were applied.

Data source and collection

Data were extracted from the EPIC electronic health record system (Epic Systems Corporation, Verona, Wisconsin, United States) using the Slicer Dicer tool. Slicer Dicer is a built-in analytics function that allows generation of aggregated, de-identified population-level data without access to individual patient identifiers. Data extraction was performed by a trained senior physician. The analysis included all eligible patients within the defined age range and study period, with no restriction on race or ethnicity.

A single Slicer Dicer query was used to identify adolescents aged 10-19 years during the study period. Data were stratified by BMI category and legal sex. BMI-for-age was categorized according to WHO growth reference percentiles as underweight (<3rd percentile), normal weight (≥3rd to ≤85th percentile), overweight (>85th to ≤97th percentile), and obesity (>97th percentile). In addition, adolescents were classified based on the presence or absence of documented BMI values in their electronic medical records.

Study variables

The primary variables extracted included BMI category, BMI measurement status, sex (male and female), and the number of patients within each category. These variables were used to describe the distribution of BMI and its relationship with sex among adolescents attending Dubai Health facilities.

Data confidentiality and ethical considerations

All data were fully de-identified prior to analysis to ensure patient confidentiality. No identifiable patient information was accessed or recorded at any stage of the study. Data were stored securely on password-protected systems accessible only to the research team. The study was reviewed and approved by the University Student and Resident Research Subcommittee of the Mohammed Bin Rashid University of Medicine and Health Sciences Institutional Review Board (approval number: MBRU IRB-2024-435).

Data analysis

Descriptive statistical analysis was performed using Microsoft Excel (Microsoft Corporation, Redmond, Washington, United States). Frequencies and percentages were calculated for BMI categories, BMI measurement status, and sex distribution across BMI groups. The study was purely descriptive, and no inferential statistical testing was performed.

## Results

A total of 496,498 adolescents aged 10-19 years attended Dubai Health facilities during 2023. Of these, 40,726 (8.20%) had a documented BMI measurement, while the vast majority, 455,772 (91.80%) had no recorded BMI value. This indicates a substantial gap in routine BMI documentation among adolescents attending healthcare services. The distribution of BMI measurement status is presented in Figure 1 and Table 1, highlighting the low rate of BMI recording within the study population despite the large number of healthcare encounters.

**Figure 1:**
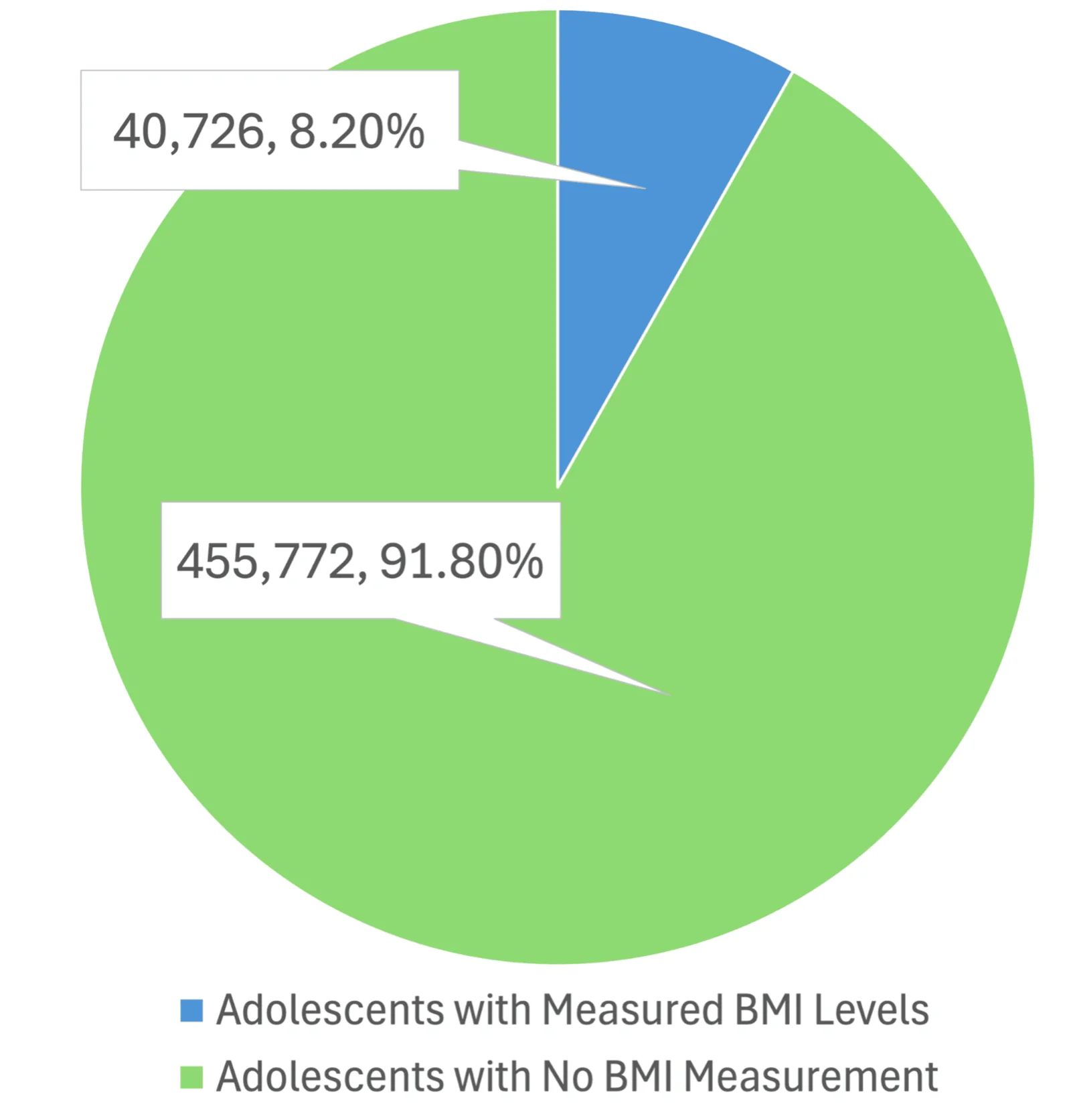
BMI measurement status among adolescents attending Dubai Health facilities in 2023

**Table 1:** BMI measurement status among adolescents attending Dubai Health facilities in 2023 (N=496,498)

Category	Frequency	Percentage
BMI Measured	40,726	8.20
BMI Not Measured	455,772	91.80

Among adolescents with available BMI data (n = 40,726), 20,442 (50.19%) were classified as having normal BMI-for-age, followed by 9,734 (23.90%) classified as obese, 7,576 (18.60%) as overweight, and 2,974 (7.30%) as underweight. Overall, 17,310 (42.50%) adolescents were classified as having excess weight (overweight or obesity), compared with 2,974 (7.30%) who were underweight, indicating a coexistence of undernutrition and excess weight within the assessed population. The distribution is presented in Table 2.

**Table 2:** Distribution of BMI categories* among adolescents aged 10–19 years with recorded BMI (N = 40,726). * classified according to WHO BMI-for-age percentiles [2]

BMI Categories	Frequency	Percentage
Underweight (<3rd percentile)	2,974	7.30
Normal (≥3rd to ≤85th percentile)	20,442	50.19
Overweight (>85th to ≤97th percentile)	7,576	18.60
Obese (>97th percentile)	9,734	23.90

Sex-based stratification of BMI categories among adolescents with recorded BMI (n = 40,726) showed broadly similar distributions between male and female adolescents across most categories. Male adolescents accounted for a slightly higher proportion of underweight (n=1,778; 59.78%) and obese (n=5,681; 58.36%), while female adolescents represented a marginally higher proportion in the normal BMI category (n=10,467; 51.20%). The overweight category was nearly evenly distributed between male adolescents (n=3,831; 50.57%) and female adolescents (n=3,745; 49.43%). Overall, no major sex-specific divergence in BMI category distribution was observed (Table 3, Figure 2).

**Table 3:** Sex distribution across BMI categories* among adolescents aged 10–19 years with recorded BMI (N = 40,726). *classified according to WHO BMI-for-age percentiles [2]

BMI Categories	Male, n (%)	Female, n (%)
Underweight (<3rd percentile)	1,778 (59.78%)	1,196 (40.22%)
Normal (≥3rd to ≤85th percentile)	9,975 (48.80%)	10,467 (51.20%)
Overweight (>85th to ≤97th percentile)	3,831 (50.57%)	3,745 (49.43%)
Obese (>97th percentile)	5,681 (58.36%)	4,053 (41.64%)

**Figure 2:**
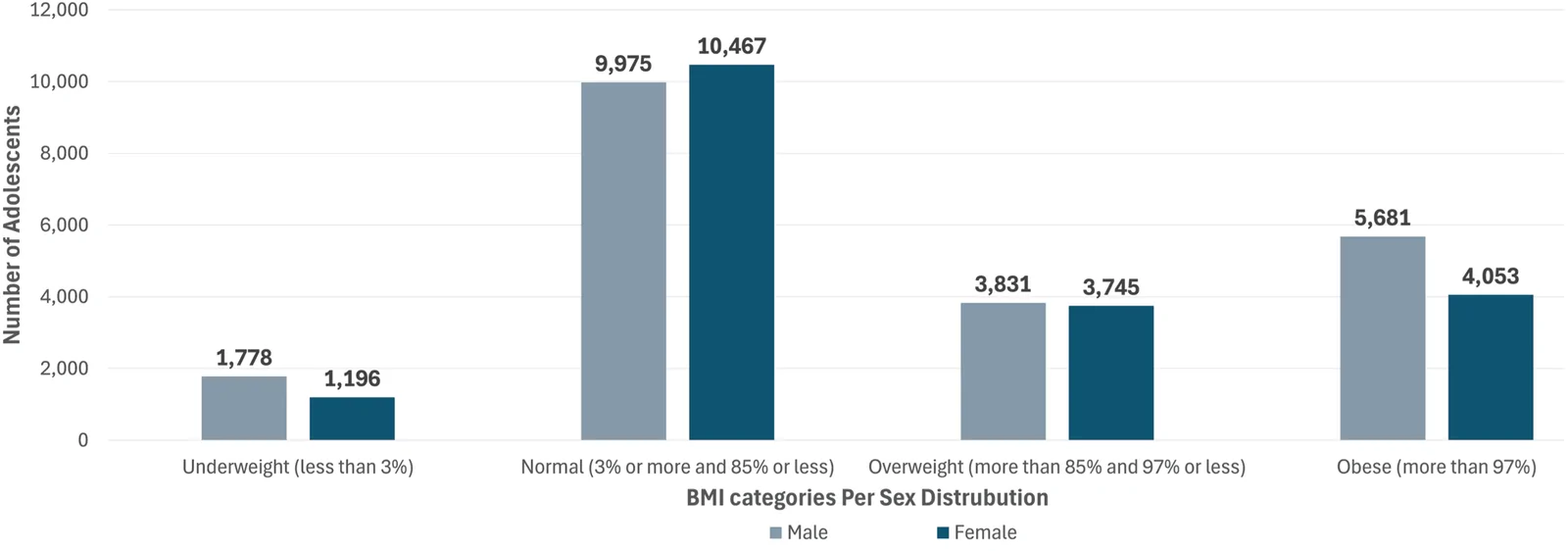
Distribution of adolescents aged 10–19 years with recorded BMI (N = 40,726) according to BMI categories by sex

Among adolescents without recorded BMI (n = 455,772), sex distribution was broadly comparable, with male adolescents accounting for 241,074 (52.90%) and female adolescents for 214,698 (47.10%). This indicates a relatively balanced sex distribution within the non-measured group, with no major sex-based disparity in missing BMI documentation.

## Discussion

Obesity is a multifactorial condition in which every component of life plays a different role. Our environment, behaviors, and genetics each hold varying degrees of importance in every individual. These components can also be divided into modifiable and non-modifiable risk factors. Some ethnicities are more likely to develop obesity due to their genetic makeup. Hispanic and Black American children were found to have a higher risk of obesity compared to their White non-Hispanic peers [5]. Studies have shown that if a mother smokes, her child is more likely to develop obesity in the first three years of life, especially when the exposure occurs between the 28th and 32nd weeks of pregnancy [7]. Children of obese mothers, even in the absence of gestational diabetes mellitus (GDM), are more likely to develop obesity [8]. As for modifiable risk factors, children living in rural areas are less likely to develop obesity than those in urban areas [9,10]. In addition, insufficient sleep is also associated with obesity among children, adolescents, and adults [11-13]. Furthermore, numerous studies have demonstrated that physical activity plays a crucial role in preventing and managing childhood obesity [14].

Obesity is a significant public health concern due to its numerous adverse health consequences. Evidence suggests that children with central adiposity are at increased risk of developing central precocious puberty compared with their non-obese counterparts [6]. In addition, obesity is commonly associated with menstrual irregularities, particularly in the context of polycystic ovary syndrome (PCOS) [15]. These obesity-related reproductive disturbances may ultimately contribute to subfertility and infertility later in life. A cross-sectional study conducted in the United States reported that adolescents who self-identified as obese were more than twice as likely to remain childless compared with their normal-weight counterparts, even after adjustment for confounding factors [16]. Additionally, increasing levels of obesity have been associated with reduced spontaneous pregnancy rates, including among women with regular ovulatory cycles [17]. Obesity is also linked to a higher risk of multiple metabolic and systemic complications, including sleep-disordered breathing, type 2 diabetes mellitus, dyslipidemia, hypertension, non-alcoholic fatty liver disease, and PCOS [18]. In the Middle East, a study conducted in Saudi Arabia by Zaki et al. demonstrated a strong association between metabolic syndrome and both abdominal and childhood obesity [19]. Moreover, childhood obesity is a well-established risk factor for the persistence of obesity into adulthood, further amplifying long-term health risks [20].

At the opposite end of the BMI spectrum, underweight adolescents also warrant significant clinical and public health attention. The primary cause is often inadequate dietary intake relative to growth requirements and resting metabolic rate, which varies among individuals [21]. Underweight status may also reflect underlying social, medical, or environmental factors [22]. Insufficient caloric and nutrient intake during adolescence can impair normal growth and development. For example, girls with anorexia nervosa have a higher prevalence of fractures compared with healthy-weight peers, largely due to reduced bone mineral density [23]. Additionally, iron deficiency and anemia are associated with impaired academic performance and increased school absenteeism, attributed to fatigue and a higher susceptibility to infectious illnesses [24]. Contrary to the common misconception that only underweight children are at increased risk of infections, evidence indicates that both underweight and obesity are associated with elevated infection risk. However, accurate assessment is often complicated by confounding factors such as hygiene, malnutrition, and underlying comorbidities [25]. From a reproductive health perspective, women with a BMI below <3rd percentile may develop functional hypothalamic amenorrhea, often related to eating disorders, excessive physical activity, or systemic illnesses associated with weight loss [26].

This study demonstrated that 7.3% of adolescents were underweight, a proportion substantially higher than that reported in a previous study conducted in Dubai in 2019, where the prevalence was 2.6% [27]. Compared internationally, the prevalence of underweight among adolescents in the present study was lower than rates reported in Brazil (8.6%), China (13.1%), and Russia (8.1%), but higher than the rate reported in the United States (3.3%) [28]. However, these comparisons should be interpreted with caution due to differences in study populations and BMI classification approaches.

In the present study, the overall prevalence of obesity was 23.9%. This prevalence is higher than that reported in a 2019 UAE-based study, which documented an obesity rate of 15.7% [27]. Similarly, a 2017 study in Ras Al Khaimah reported a lower prevalence of 22.7% [20]. International comparisons also demonstrate higher obesity rates among adolescents in several countries, including the United States (18.4%) [4], Kuwait (14.6%) [29], and Iraq (22.3%) [30]. In contrast, a lower prevalence was reported in China (10.5%) [31]. These observed variations in obesity prevalence may be attributed to differences in ethnicity, socioeconomic status, dietary patterns, and levels of physical activity across populations.

In this study, 18.6 % of adolescents were classified as overweight, which is higher than the 14.5% reported in a study conducted in Ras Al Khaimah in 2017 [20], and lower than another study conducted in the UAE, which reported that 25.3% of cases were overweight [27]. Notably, a substantial proportion of adolescents in our cohort (50.2 %) had a normal BMI, a finding that is important for descriptive characterization of the screened population and not the overall adolescent population.

Adolescents in the UAE come from diverse ethnic backgrounds, and parental and cultural practices may significantly influence BMI levels. Health education campaigns in schools are essential to raise awareness about obesity and its negative health impacts. Within the school setting, engaging activities such as sports days and healthy cooking workshops can promote positive lifestyle behaviors. Furthermore, establishing a system to identify adolescents at risk of obesity and provide ongoing surveillance is crucial. Continuous monitoring of the effectiveness of these health strategies is necessary, and alternative interventions should be considered if current approaches are insufficient.

In our study, 91.8% of adolescents attending Dubai Health facilities did not have their BMI measured, highlighting the urgent need for healthcare providers to implement routine BMI assessment and monitoring. This low documentation rate may reflect variability in clinical workflows and encounter types, where anthropometric measurements are not consistently prioritized, particularly in acute or problem-focused visits or administrative encounters. In addition, the absence of automated electronic health record prompts for BMI calculation may further contribute to under-documentation.

To address this gap, electronic health record-based interventions may be implemented, including automated prompts or mandatory fields for BMI entry during adolescent encounters. Furthermore, integrating clinical decision support tools, such as linking medication prescribing or order completion to the documentation of key anthropometric parameters (including weight and height), may improve completeness of BMI recording and strengthen adherence to standardized screening protocols.

Adolescent BMI is a strong predictor of health in adulthood. Obesity during adolescence is a known risk factor for persistent obesity in adulthood, contributing to increased morbidity and mortality, including a higher risk of type 2 diabetes, hypertension, and non-alcoholic fatty liver disease [5]. Conversely, low BMI and malnutrition can have serious consequences on growth and development, increasing the risk of hair loss, hypothermia, and menstrual disorders in adolescent girls [3]. Additionally, both underweight and overweight adolescents may experience disturbances in body image and perception, which can escalate into body dissatisfaction and potentially contribute to the development of eating disorders.

Limitations

This study has several limitations. While the use of electronic health records enhanced data accuracy and reduced reporting bias, it limited the availability of detailed contextual information. Important potential confounders, including lifestyle behaviors, dietary patterns, physical activity levels, medication use, and comorbid conditions, were not captured and may have influenced the observed results. Additionally, BMI measurements were obtained at a single time point, which precluded assessment of temporal trends or changes in BMI over time. A major limitation of this study is the substantial proportion of adolescents (91.8%) who did not have BMI measurements recorded, which may introduce selection bias and limit the generalizability of BMI distribution findings to the overall adolescent population. Furthermore, BMI-for-age categories were derived from pre-classified outputs within the EPIC Slicer Dicer system; the exact internal algorithm (e.g., Z-score or percentile computation method) was not accessible, which may limit reproducibility in other electronic health record systems or analytical platforms. Future studies incorporating longitudinal data and qualitative approaches, such as participant interviews, may provide a more comprehensive understanding of the determinants of adolescent BMI.

## Conclusions

BMI remains a key indicator of adolescent health and provides important insight into both undernutrition and overnutrition within a population. In this study, approximately half of the adolescents with recorded BMI measurements had a normal BMI, while 7.30% were underweight, 18.60% were overweight, and 23.90% were obese, reflecting a dual burden of malnutrition in this population. In addition, a substantial proportion of adolescents (91.8%) did not have BMI measurements recorded during healthcare encounters, highlighting a significant gap in routine anthropometric screening within clinical practice. These findings underscore the importance of strengthening BMI assessment as part of routine adolescent healthcare, alongside early identification and targeted interventions for both undernutrition and excess weight. Integrating preventive strategies focused on nutrition education and physical activity may contribute to improving long-term health outcomes and reducing future disease burden in this population. Future quality improvement initiatives are planned within Dubai Health facilities to re-evaluate BMI documentation practices following implementation of electronic health record enhancements, in order to assess their impact on improving screening rates and adolescent BMI monitoring over time.
